# Inter- and Intraspecific Identification of the Screwworm, *Cochliomyia hominivorax*, Using Random Amplified Polymorphic DNA-Polymerase Chain Reaction

**DOI:** 10.1673/031.013.7601

**Published:** 2013-08-10

**Authors:** Steven R. Skoda, James L. Figarola, Saowaluck Pornkulwat, John E. Foster

**Affiliations:** 1 USDA-ARS-KBUSLIRL-Screwworm Research Unit, 2700 Fredericksburg Rd., Kerrville, TX 78028; 2 Beckman Research Institute of the City of Hope National Center, Duarte, California 91010; 3 FMC Chemical, Southeast Asia Zone Development Manager, Thailand; 4 Department of Entomology, University of Nebraska, Lincoln, Nebraska, USA 68583-0816

**Keywords:** genetic markers, myiasis, sterile insect technique

## Abstract

The screwworm, *Cochliomyia hominivorax* (Coquerel) (Diptera: Calliphoridae), is one of the most devastating arthropod pests of livestock in the Western Hemisphere. Early instars are very difficult to distinguish morphologically from several closely related blow fly species. Random amplified polymorphic DNA-polymerase chain reaction (RAPD-PCR) markers were developed for identifying *C. hominivorax* from other wound inhabiting species. Forty decameric primers were screened; nine showed clear reproducible RAPD profiles suitable for distinguishing all life stages of *C. hominivorax* from 7 other species, including *C. macellaria* (Fabricius). The results from RAPD-PCR with field-collected samples of unknown first instars agreed with morphological identification that the samples were not *C. hominivorax*. Three different primers showed DNA polymorphisms (intraspecific) for samples originating from Mexico, Costa Rica, Panama, Jamaica, and Brazil. Therefore, RAPD-PCR may be useful for determining the geographic origin of *C. hominivorax* samples. Comparing products from these primers, used with known and unknown screwworm samples from an outbreak in Mexico, clearly showed that the outbreak did not originate from the mass rearing facility. Accurate identification of suspected *C. hominivorax* samples is possible using RAPD-PCR. Further development to identify the geographic origin of samples would benefit the ongoing surveillance programs against *C. hominivorax* and the decision process during suspected outbreaks of this important pest.

## Introduction

The screwworm, *Cochliomyia hominivorax* (Coquerel) (Diptera: Calliphoridae), is an obligate parasite of warm-blooded animals. Larvae feed on living tissues, causing a condition called myiasis (James 1947). *C. hominivorax* was one of the most devastating pests of livestock in North America before successful eradication using the sterile insect technique (Vargas-Terán et al. 2005). Screwworms remain a threat to humans and domestic animals in currently endemic regions of South America and the Caribbean. Introduction and subsequent eradication of *C. hominivorax* to new regions or to previously eradicated regions are well documented (Spradberry 1994). There is an ongoing threat of introduction to the U.S., other regions where *C. hominivorax* has been eradicated, and areas where it is not endemic.

Effective monitoring, surveillance, and quarantine measures against *C. hominivorax* depend on accurate and timely identification. However the morphologically similar secondary screwworm, *Cochliomyia macellaria* (Fabricius), co-exists with *C. hominivorax*, resulting in numerous misidentifications (particularly for first and second instars) using traditional morphological methods. Other flies in the families Calliphoridae and Sarcophagidae are attracted to necrotic wounds of animals (Hall and Wall 1995), further confounding the difficulties of correct identification, especially for the early instars. Additionally, the possibility of sabotage or fraud exists for any samples suspected to be *C. hominivorax* but in question (*e.g*. 1992–1993 Mexican outbreak), making it difficult for program managers to respond appropriately. Consequently, emergency releases of sterile flies have been made, costing millions of dollars, even on samples that proved negative on later analysis (Anonymous 1990). Thus, identification methods that are accurate, timely, and potentially capable of indicating the origins of *C. hominivorax* samples would be an important addition to the current eradication and barrier maintenance program against *C. hominivorax*.

Techniques in molecular biology have provided powerful tools for discriminating species and populations using molecular markers (Loxdale and Lushai 1998; Semagn et al. 2006; Pereira et al. 2008; Samarakoon et al. 2012), including applications with screwworms (Azeredo-Espin and Lessinger 2006; Alamalakala et al. 2009). One technique useful for inter- and intraspecific identification is the random amplified polymorphic DNApolymerase chain reaction (RAPD-PCR) (Semagn et al. 2006). RAPD-PCR utilizes short, synthetic oligonucleotides of random sequences as a single primer that is able to anneal and prime at multiple locations throughout the genome of an organism; a spectrum of amplification products are pro-duced that are characteristics of the template DNA (Welsh and McClelland 1990; Williams et al. 1990). The presence and absence of a specific PCR product is diagnostic for the oligonucleotide-binding sites on genomic DNA (Williams et al. 1995) and, therefore, can serve as useful molecular markers for taxonomic and population genetic studies (Semagn et al. 2006; Feng et al. 2009). Compared with other DNA-based methods, the advantages of RAPD-PCR include the quick speed and ease with which results can be obtained, relatively low cost, small DNA sample requirements, and the ability to identify hundreds of new markers in a short time (Hadrys et al. 1992; Semagn et al. 2006). Additionally, no preliminary knowledge of the subject genome is necessary, therefore eliminating the requirements for isolation of cloned DNA probes, preparation of filters for hybridization, and nucleotide sequencing (Jain et al. 2010). Thus, RAPD-PCR can be performed in a moderately equipped laboratory for most applications.

RAPD-PCR has been used to identify cryptic, sibling, and related species of mosquitoes (Ballinger-Crabtree et al. 1992; Wilkerson et al. 1993; Sucharit and Komalamisra 1997), honey bees (Suazo et al. 1998), black flies (Duncan et al. 2004), and screwworms (Skoda et al. 2002). Molecular markers generated by RAPD-PCR were useful in determining the geographic origins of a weevil (Williams et al. 1994), stored product moth (Dowdy and McGaughey 1996), gypsy moth (Schreiber et al. 1997), and fruit flies (Reyes and Onchado 1998), and in estimating gene flow and genetic variability in *C. hominivorax* (InfanteMalachias et al. 1999; Azeredo-Espin and Lessinger 2006) as well as several species of medically-important mosquitoes (de Sousa et al. 2001; Gonzáles et al. 2007; Hiragi et al. 2009). Additionally, inter- and intraspecific differentiation by RAPD-PCR has been applied to the Mediterranean fruit fly (Sonvico et al. 1996), triatomine bugs (Garcia et al. 1998), and horn flies (Castiglioni et al. 2005), with the capability of identifying biotypes or ecotypes/ecoraces of various insect species (Guirao et al. 1997; Pornkulwat et al. 1998; Saha and Kundu 2006).

Here, our objective was to further develop RAPD-PCR for accurate and timely identification of *C. hominivorax* from other wound inhabiting flies, and to gain insight into the potential of RAPD-PCR to discriminate geographic origin of screwworm samples.

## Methods and Materials

### Insect specimens

Field collected samples from Brazil (adults and third instars) and the Mexico outbreak (all third instars) were maintained at -70° C until needed. Field collected larvae from a suspected outbreak in Nicaragua (first and early second instars) were initially stored in 95% ethanol; on arrival to our facility, these samples were placed at -20° C. All other *C. hominivorax* were obtained from laboratory colonies, 9 strains in all, maintained at the USDA-ARS Midwest Livestock Insects Research Unit-Biosecure Screwworm Rearing Laboratory in Lincoln, Nebraska, USA (Table 1). Colonies of *C. macellaria, Chrysomya rufifacies* (Macquart), *Calliphora vicina* (Robineau-Desvoidy), *Phormia regina* Meigen, *Lucilia sericata* Meigen, *Sarcophaga* sp. Meigen (Sarcophagidae), and *Musca domestica* L. (Muscidae), were established from flies collected at blood and liver baited traps outside the laboratory (Table 1). All samples of life stages from colony flies were frozen and stored at -70° C prior to use. Additional samples of *C. macellaria* adults, collected from 3 locations in Jamaica in 1998, were maintained in 95% ethanol (Table 1). Samples from Mexico, a confirmed outbreak of suspicious origin, included: 1) larvae of known origin, collected and labeled as from the mass production colony, for comparison to unknowns, and 2) unknown samples (both field collected and from the mass production colony); origins of unknowns were not revealed to the authors (a blind test).

**Table 1.**
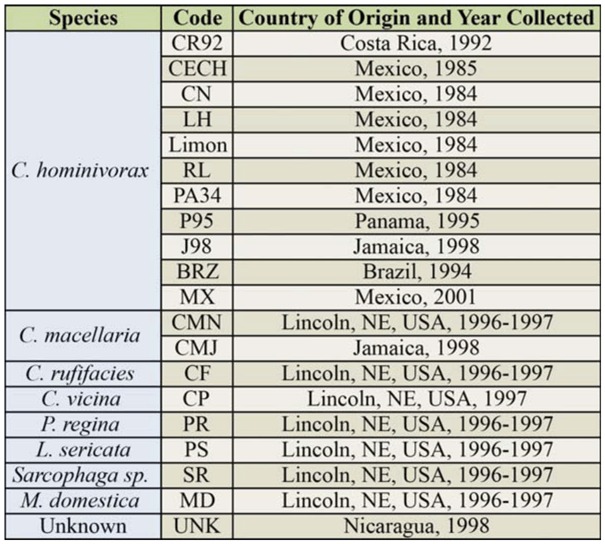
Code, origin, and year of collection of specimens of *Cochliomyia hominivorax* and related flies used for RAPD-PCR.

### DNA extraction

All chemicals for DNA extraction were from Sigma-Aldrich (www.sigmaaldrich.com) unless otherwise noted. DNA was extracted from single individuals using only head capsules in the case of the older larvae and pupae, and heads or legs of newly-emerged adults, to minimize contamination and preserve taxonomic value of specimens. For eggs and first instars (< 24 hr old), ∼10 mg and 5 individuals, respectively, were used per DNA sample. The samples were ground in 1.5 mL microcentrifuge tubes, and total genomic DNA from individual samples was extracted by a modified cetyl ammonium bromide method (Rogers and Bendich 1985) using 400 µL of cetyl ammonium bromide buffer (100 mM Tris-HCl, 1.4 M NaCl, 0.02 M EDTA, 2% cetyl ammonium bromide, and 0.2% &Bgrlmercapto ethanol added just before use) and incubating the samples with 10 µL of Proteinase K (20 mg/mL) and 5 µL of RNAse (50 mg/mL) at 55° C and 37° C for 1 and 2 hr respectively. Additionally, phenol-chloroform extraction was performed once, after which DNA was precipitated with ice-cold 95% ethanol. After precipitation in 400 µL of chilled isopropanol (-20° C for > 2 hr) followed by centrifugation for 30 min in a cooled microcentrifuge (4° C; 14,000 rpm), supernatant was discarded, and DNA pellets were resuspended with 50 µL of Tris-EDTA buffer. The DNA concentration was estimated by running an aliquot in 1% agarose gel (in 1x TBE buffer) with a known concentration of Lambda Hind III marker (Gibco-BRL, Invitrogen, www.invitrogen.com). Aliquots of the resuspended pellet were diluted 5–20 fold (depending on the concentration) to yield a final DNA concentration of ∼10 ng/µL for use in RAPD-PCR.

### Polymerase chain reaction

PCRs were performed using the Perkin-Elmer GeneAmp® PCR 9600 thermocycler and reagents (www.perkinelmer.com). The RAPD protocol described here was adapted from Pornkulwat (1998) using an optimum template concentration of ∼10 ng. Reaction mixtures of 25 µL total volume per tube contained 12.2 µL of sterile distilled water; 1 µL of 1% Nonidet P-40; 2.5 µL of 10x Stoffel buffer; 4 µL of 25 mM MgCl_2_; 0.75 µL, each of 10 mM dCTP, dATP, dGTP, and dTTP; 1 µL of 10 pmole 10-mer primer (Operon Technologies, Qiagen, www.qiagen.com); 1 µL of diluted DNA from individual samples; and 0.3 µL of AmpliTaq® DNA Polymerase Stoffel Fragment (Invitrogen). A master mix of all the PCR components, except the template DNA, was made, aliquoted to the appropriate tubes, and then the DNA sample was added. Negative controls included all reaction components, except template DNA, which was replaced with an equal volume of sterile distilled water.Forty decameric primers were used for initial screening (OPA-7, OPA-12, OPA-16, OPB-1, OPB-10, OPB-13, OPB-17, OPD-16, OPD-20, OPE-1 to OPE-20, OPG-5, OPG-6, OPG9, OPG-10, OPG-1, OPI-10, OPI-11, OPI-15, OPI-16, OPJ-8, and OPJ-11).

The following temperature conditions were used: 95° C for 1 min, 94° C for 1 min, 36° C for 30 sec, and 72° C for 1 min (10 cycles); 94° C for 10 sec, 35° C for 30 sec, and 72° C for 30 sec (30 cycles); and a 72° C extension step for 5 min. Eight µL, of RAPD products for individual samples were loaded and electrophoresed in 1.5% agarose gel (in 1x TBE buffer) with DNA molecular size standards (Gibco-BRL) and a negative control at 60 V for about 2 hours. After electrophoresis, gels were stained with ethidium bromide for 15 min and photographed over a UV transilluminator.

### Data Analyses

For screening of species-discriminating markers, DNA was extracted from 10 individual adult flies per strain and species and 3 individuals per immature stage. Eight adult flies per population of *C. hominivorax* were used individually for screening of intraspecific markers. Primers that generated too many bands were eliminated, and only those that produced clear, distinct, and reproducible bands in all life stages were considered. Two replicates, using the same number of individuals as in screening, were done each for interand intraspecific analyses. At least 3 PCR repetitions per DNA sample were performed, and 2 batches of primers were used to ensure reproducibility of results. Photographs of agarose gels were scanned by HP Scan Jet IIc (Hewett-Packard, www.hp.com), and the sizes of RAPD generated bands were estimated using the DNA ProRLFP® program (DNA ProScan, Nashville, TN). In all RAPD numerical analyses, band size tolerance (the percentage a band can deviate on either side of the size value and still be considered a match) was set at 1–3%.

Genetic distances were calculated, using all scorable bands (presence/absence) from all selected RAPD primers, with the procedure of Nei and Li (1979) using PAUP® (Swofford 2002). Bootstrapping (1000 replicates) was used to determine support for dendrograms that were derived by the unweighted pairgroup method using arithmetic averages using PAUP^®^ (Swofford 2002).

## Results

### Species discrimination

**Figure 1.**
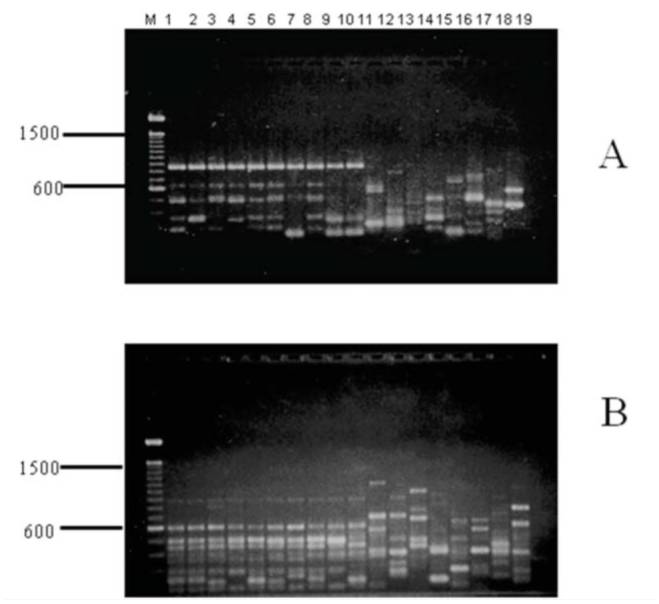
Representative RAPD profiles for individuals of *Cochliomyia hominivorax* and other fly species with primer (A) OPA-12 and (B) OPE-14. *C. hominivorax* are in lanes: (1) CR92 (Costa Rica), (2) CECH (Mexico), (3) CN (Mexico), (4) LH (Mexico), (5) LI (Mexico), (6) RL (Mexico), (7) PA34 (Mexico), (8) P95 (Panama), (9) J98 (Jamaica), (10) BRZ (Brazil); other species are (11) CMN (*C. macellaria*), (12) CMJ (*C. macellaria*), (13) CF (*Chrysomyia rufifacies*), (14) CP (*Calliphora vicina*), (15) PR (*Phormia regina*), (16) PS (*Lucilia sericata*), (17) SR (*Sarcophaga sp*.), (18) MD (*Musca domestica*), (19) control. Lane M contains 100 bp ladder markers. High quality figures are available online.

**Figure 2.**
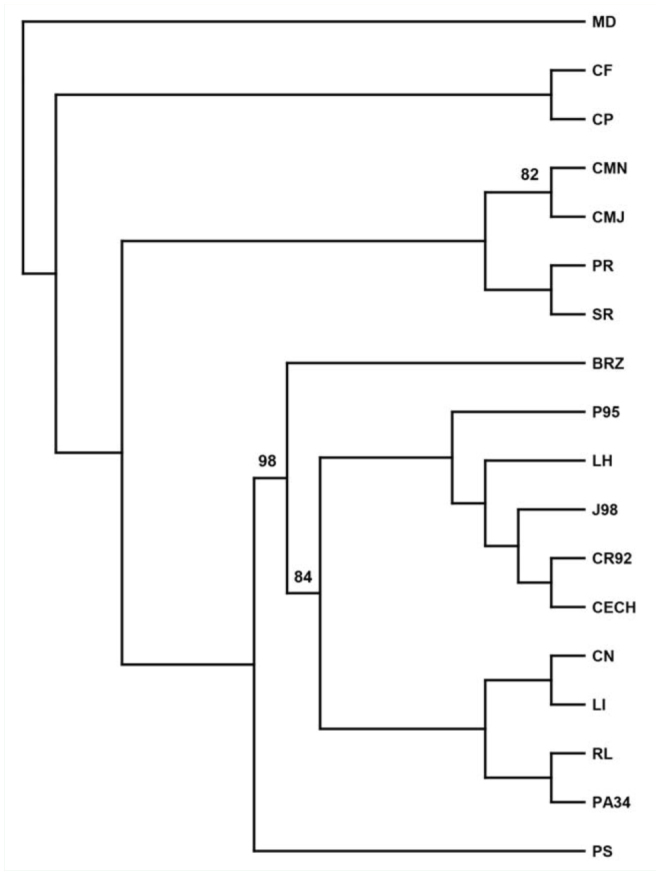
Dendogram of the relationships between 10 populations of *C. hominivorax* CR92 (Costa Rica), CECH (Mexico), CN (Mexico), LH (Mexico), LI (Mexico), RL (Mexico), PA34 (Mexico), P95 (Panama), J98 (Jamaica), BRZ (Brazil) and other closely related myiasigenic flies CMN (*C. macellaria*), CMJ (*C. macellaria*), CF (*Chrysomyia rufifacies*), CP (*Calliphora vicina*), PR (*Phormia regina*), PS (*Lucilia sericata*), SR (*Sarcophaga sp*.), MD (*Musca domestica*) using all gels and all scorable RAPD bands amplified by primers OPA-12, OPA-16, OPB-1, OPE-3 and OPE-16. Similarity values calculated by Nei-Li's coefficient; UPGMA clustering; only bootstap values > 50% reported. High quality figures are available online.

PCR amplification of DNA from *C. hominivorax* and related flies by random primers resulted in the production of discrete banding profiles for all primers tested. Primers were selected based on patterns that generated consistent and reproducible diagnostic results for *C. hominivorax*. To identify a species-specific primer, some or a combination of amplified DNA fragments must be unique to *C. hominivorax*, and should be present in all individuals and life stages of the species. Of the 40 decameric primers initially screened, 9 gave RAPD patterns that distinguished the 10 populations of screwworms from the other fly species (Table 2) by a single diagnostic band (Figure 1a) or a combination of bands (Figure 1b). Few co-migrating bands were shared across the different fly species, demonstrating that screwworm samples can be readily identified on the basis of their RAPD banding patterns with any one of the nine primers. Additionally, cluster analyses using all scorable RAPD bands for each of the 9 primers correctly grouped and separated all 10 *C. hominivorax* populations from the other fly species; the screwworm ‘fork’ exhibited 98% bootstrap support (Figure 2). However, the 5 geographic populations (Costa Rica, Mexico, Panama, Jamaica, and Brazil) of *C. hominivorax* could not be differentiated using these 9 primers.

**Table 2.**
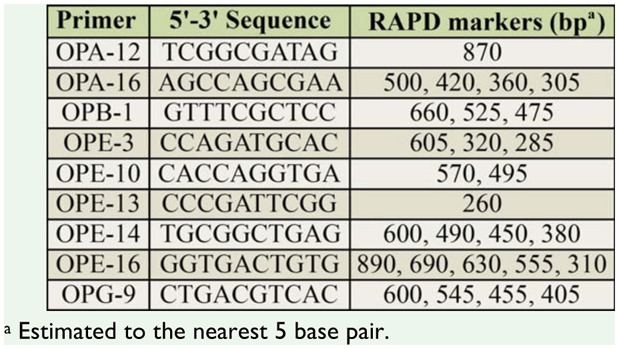
Nine species-specific primers, their sequences, and their respective RAPD markers diagnostic of *Cochliomyia hominivorax*.

**Figure 3.**
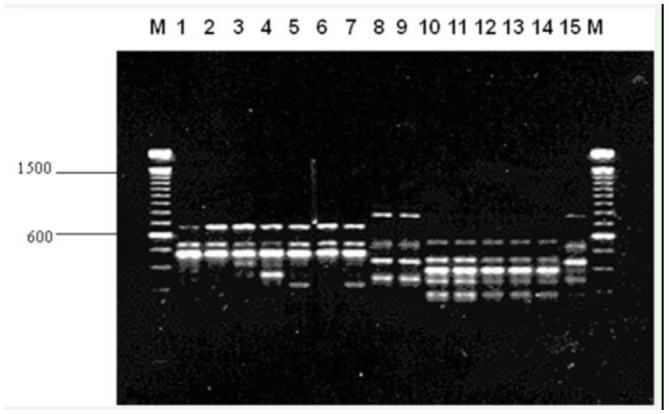
Representative gel comparing RAPD profiles between individuals of different stages of *Cochliomyia hominivorax* (lanes 1–7) with *C. macellaria* (lanes 10–15) using primer OPB-1. Lanes: (1–2) (CR92; Costa Rica) eggs, (3–4) first instar, (5) third instar, (6) pupa, (7) adult, (8–9) *C. macellaria* (CMN; Nebraska) eggs, (10–11) first instar, (12) third instar, (13–14) pupa, (15) adult. Lane M contains 100 bp ladder markers. High quality figures are available online.

The diagnostic RAPD bands or banding patterns produced by these speciesdiscriminating primers were consistently generated in all life stages of the fly. Despite some variations from individuals within a species and stage, the identified RAPD diagnostic markers were present in all life stages of *C. hominivorax*, and the banding patterns clearly differed with those of immature *C. macellaria* (Figure 3).

**Figure 4.**
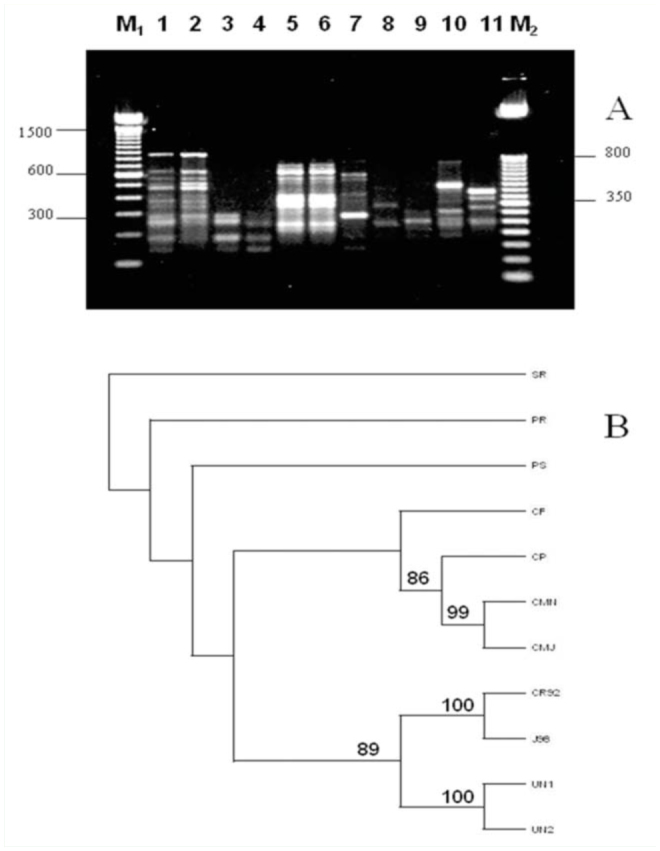
(A) Representative gel comparing RAPD profiles of individual unknown first instars from Nicaragua with first instars of *Cochliomyia hominivorax* in lanes (1) CR92 (Costa Rica) and (2) J98 (Jamaica) and other flies using primer OPA-12 (3–4) CMN (*C. macellaria*), (5–6) UNK, (7) CF , (*Chrysomyia rufifacies*) (8) CP (*Calliphora vicina*), (9) PR (*Phormia regina*), (10) PS (*Lucilia sericata*), (11) SR (*Sarcophaga sp*.). Lanes M1 and M2 contain 100 bp and 50 bp ladder markers, respectively. (B) Dendogram of the relationships between unknowns (UNK) from Nicaragua with screwworms and other flies from all gels and all scorable RAPD bands amplified from primer OPA-12, OPB-1, OPE-3 and OPE-14. Abbreviations same as for A above. Similarity values calculated by Nei-Li's coefficient; unweighted pair-group method using arithmetic averages clustering; only bootstap values > 50% reported. High quality figures are available online.

Five larvae of unknown first instars collected from cattle wounds in Nicaragua were analyzed together with the same number of larvae of *C. hominivorax* and the other fly species. The unknown insect was not *C. hominivorax* (Figure 4a). Morphological identification placed the sample as *Phaenicia* *(*=*Lucilia)*. However, RAPD patterns for *L. sericata* samples from Nebraska did not match (Figure 4a). Cluster analyses using the unweighted pairgroup method indicated that the unknown was closer to *C. hominivorax* than *L. sericata* or the other species (Figure 4b).

**Table 3.**
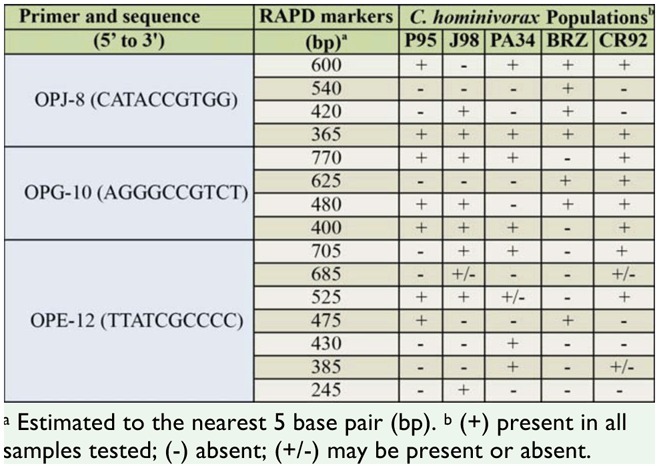
Potential intraspecific RAPD primers for identification of *Cochliomyia hominivorax* populations.

### Variation among *C. hominivorax* populations

**Figure 5.**
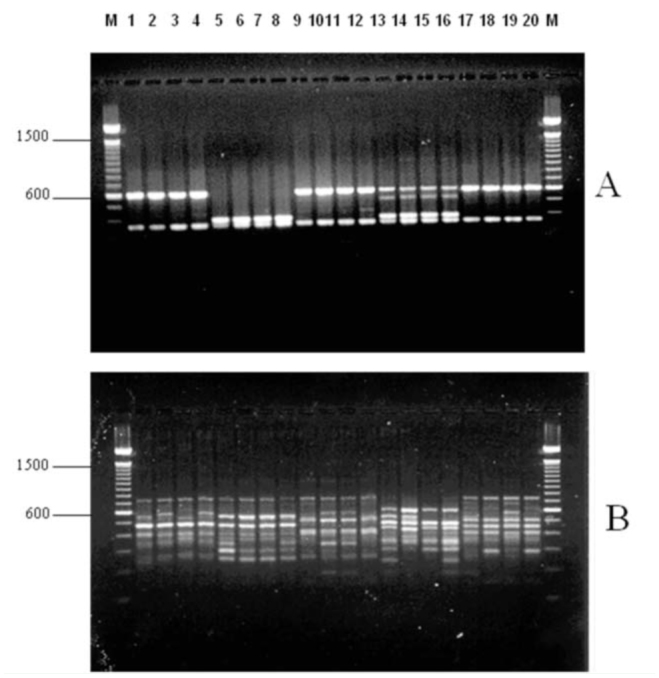
Representative gels of RAPD profiles with primer OPJ-8 (A), and OPG-10 (B) using individuals of *Cochliomyia hominivorax* originating from 5 different locations. Lanes: (1–4) P95 (Panama), (5–8) J98 (Jamaica), (9–12) PA-34 (Mexico), (13–16) BRZ (Brazil), (17–20) CR92 (Costa Rica). Lane M contains 100 bp ladder markers. High quality figures are available online.

RAPD profiles of individual adults of wild type populations of *C. hominivorax* from Costa Rica, Mexico, Jamaica, Panama, and Brazil were compared with 3 primers initially identified for potential strain-specific markers. At least 15 potential intraspecific RAPD markers were generated from these 3 primers (Table 3). Discrimination of *C. hominivorax* populations based on the presence/absence of some RAPD-PCR products was possible. For example, using primer OPJ-8, the presence of ∼600, 540, 420, and 365 bp products distinguished the *C. hominivorax* populations from Brazil, while a combination of 420 and 365 bp was diagnostic of Jamaican populations (Figure 5a; Table 3). OPG-10 produced several bands, of which the absence of ∼770 bp and 480 bp fragments was diagnostic of Brazilian and Mexican *C. hominivorax* populations, respectively (Figure 5b; Table 3). In contrast, OPE-12 produced at least 7 bands that could potentially differentiate all the *C. hominivorax* populations that were analyzed (Table 3).

**Figure 6.**
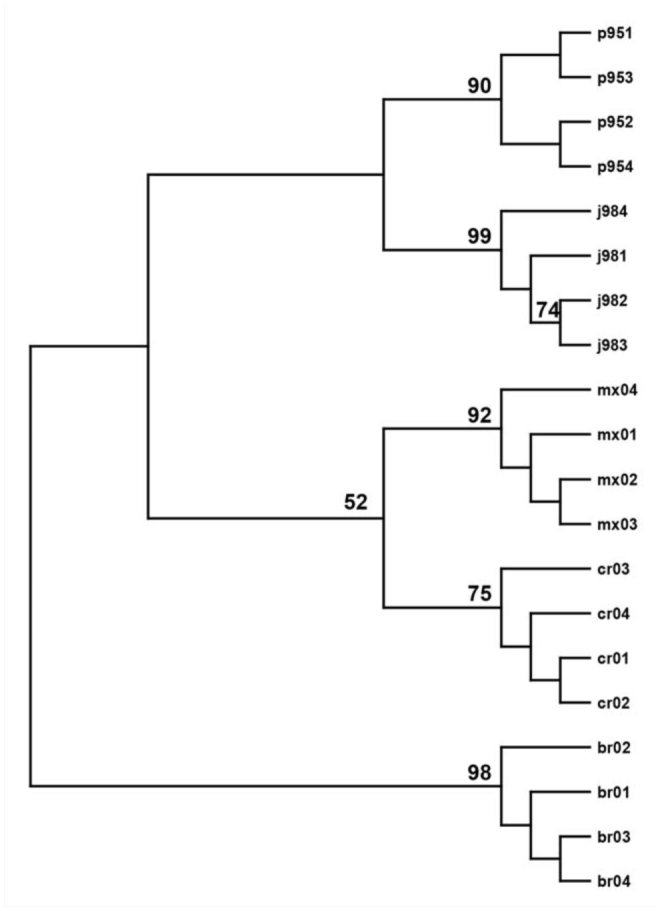
Dendogram of the relationships between *Cochliomyia hominivorax* from 5 geographic locations using all gels and all scorable RAPD bands amplified by 3 primers (OPE-12, OPG-10, OPJ-8); P95 = Panama, J98 = Jamaica, MX = Mexico, CR = Costa Rica, BR = Brazil. Similarity values calculated by Nei-Li's coefficient; unweighted pair-group method using arithmetic averages clustering; only bootstap values > 50% reported. High quality figures are available online.

Numerical analyses of RAPD-PCR products from the 3 primers (OPG-10, OPE-12, and OPJ-8) resulted in clear-cut population grouping of each fly sample according to its geographical origin (Figure 6). Analysis of RAPD patterns showed strong support for each population ‘branch.’ Overall cluster analysis using all scorable RAPD bands generated from the 3 primers suggests that flies from Brazil form an independent group separate from the other 4 populations. *C. hominivorax* from Costa Rica were more closely associated with the Mexican populations while Panama and Jamaica *C. hominivorax* clustered together (Figure 6).

The same 3 primers used to detect intraspecific variation, along with OPA-12 and OPI-10, were used with samples from the Mexico outbreak. The results, including photographs of gels and dendrograms generated with unweighted pair-group analysis, were sent to the sample curator (who designated the field vs. colony unknowns). The pattern of RAPD amplification products for the field-collected outbreak samples did not match that from the mass production facility, as evidenced from gel images and dendrograms (data not shown); therefore, sabotage from the facility for mass production of screwworms, as originally feared/suspected, was ruled out.

## Discussion

Several studies have shown that the number of loci amplified with RAPD primers depends on factors such as reagents and reaction conditions, sample conditions, and DNA quality and extraction methods (Williams et al. 1990; 1995; Black 1993; Ellsworth et al. 1993; Kernodle et al. 1993; MacPherson et al. 1993; Meunier and Grimont 1993; Micheli et al. 1994; Gallego and Martinez 1997; Khandka et al. 1997). Variations were minimized and amplifications of artifacts in the RAPD profiles were prevented by using heads and legs of individual frozen flies, by using similar amounts of template DNA for PCR runs, by running a negative control in each reaction, and by using 2 batches of each potential primer and AmpliTaq® DNA Polymerase Stoffel Fragment during the replication process. Stoffel Fragment is a 61 kDa modified form of recombinant AmpliTaq® DNA Polymerase from which the N-terminal 289 amino acids have been deleted to increase stringency at lower ionic strength and reduce misextension. The use of Stoffel Fragment is very important for reproducibility of results, a negative attribute often attributed to RAPDs (Pereira et al. 2008). Additionally, amplification conditions that previously resulted in reproducible RAPD profiles were used (Pornkulwat 1998; Skoda et al. 2002). Primer selection was based on discrete and reproducible fragments, i.e., those that produced a smaller number of intense, diagnostic bands, as seen on agarose gels stained with ethidium bromide. Reproducible genetic markers were identified from the decameric primers used for species-specific and intraspecific identifications. Sample identification can de done in < 10 hr, starting from DNA extraction to actual visualization of amplified products on agarose gels.

The diagnostic bands for each of the 9 primers suitable for *C. hominivorax* identification were selected based on their reproducibility and consistency of amplification products in all *C. hominivorax* life stages. In addition, all diagnostic markers were present in all 10 *C. hominivorax* populations analyzed in this study. The diagnostic marker can be a single RAPD band (e.g., OPA-12, OPE-13), or a combination of bands (OPB-1, OPE-3, OPE-14, OPE-16, OPG-9). In practice, we recommend that several primers should be used for accurate diagnosis of samples, especially for poorly-preserved specimens. For example, a loss of some marker bands was observed from samples preserved in 70% alcohol for more than 3 months (S. Pornkulwat, personal observation) compared with 95% ethanol, such that identification through amplification products from a single primer may not be reliable. Similar findings have been reported for *L. sericata* (Stevens and Wall 1995). We further recommend that samples be preserved in 95% ethanol (if alcohol is the only storage medium and for long-term storage) for this RAPDPCR method to be accurate and reliable.

The accuracy of this technique for identifying screwworms was evaluated when unknown Nicaraguan samples of first instars taken from myiasis wounds were analyzed. Although Nicaragua had been declared screwworm-free since 1997, the possibility of reintroduction in this country due to livestock trade and movement is always present. RAPD-PCR of DNA from the unknown showed that the sample was not *C. hominivorax*. This conclusion was supported by results from morphological identification. The unknown was identified as *Lucilia* sp. based on available keys, but RAPD banding patterns of the *L. sericata* collected from Nebraska were different from the unknowns. Interestingly, cluster analyses indicated that the unknowns were closely related to *C. hominivorax*. Polymorphisms have been shown to exist among populations of *L. sericata* worldwide (Stevens and Wall 1997), and probably all species of flies from different geographical areas, such that identification of negative samples by RAPD-PCR is impractical, if not impossible at this time, based on the limited genetic markers for these non-screwworm species. Nevertheless, as noted above, all the RAPD diagnostic markers generated from all 9 species-specific primers were always present in all 10 *C. hominivorax* populations used here; these populations originated from distinctly different geographic regions. Additionally, results from cluster analyses of RAPD banding patterns for each of the 9 species-discriminating primers, strongly supported by bootstrapping analyses, resulted in the correct separation of *C. hominivorax* samples from non-screwworm specimens (using 1–3% band size tolerance matches). This further indicates the utility of RAPD-PCR for identification and emphasizes the need to expand the molecular genetic database in order to identify which species is present if samples are identified as not being *C. hominivorax*. The possibility of using some of these RAPD markers (particularly the single RAPD marker from OPA-12 and OPE-13) as species-specific DNA probes for *C. hominivorax* and their potential for field use should be investigated. Such RAPD-derived probes, sequence characterized amplified regions (SCAR), have been successfully used to identify related bacterial (Argenton et al. 1996; Oakey et al. 1998), protozoan (trypanosome) (Oury et al. 1997), silkworms (Saha and Kundu 2006), and other species.

Although not the main focus, analyses of *C. hominivorax* samples originating from 5 geographic locations using RAPD-PCR suggested that this technique may be able to discriminate between these populations. Genetic analysesbased on pair-wise comparisons of RAPD bands showed that intraspecific genetic variation existed within and among these populations, in general agreement with results from earlier studies (Infante-Malachias et al. 1999; Skoda et al. 2002; Azeredo-Espin and Lessinger 2006). However, all individuals within a population were observed to cluster, suggesting that this technique may be suitable for molecular fingerprinting and possible identification of the geographical origin of a *C. hominivorax* sample. The analyses using all scorable RAPD bands generated by the 3 primers indicated that flies from Brazil form separate populations different from those of Jamaica, Mexico, and Central America. This finding was also indicated in the dendrogram generated from data of RAPDs using the primers for identifying screwworms from other species. These results differ somewhat from the conclusions made by Taylor et al. (1996), who divided *C. hominivorax* populations into 3 assemblages (North and Central America, South America, and Jamaica) but were using RFLP of mtDNA and had fewer samples and fewer bands for analysis.

The data presented here provide useful molecular markers only for possible identification of the geographical origins of the *C. hominivorax* sample. The results are still preliminary and should not be regarded as indicators of the level of genetic variability or relationships among various *C. hominivorax* populations. It would be necessary to analyze several more *C. hominivorax* samples from South America and the Caribbean to develop more comprehensive genetic ‘fingerprints’ for later comparisons with suspected *C. hominivorax* infestation samples. Obviously, much greater diversity might be found if specimens representing the entire geographical range of *C. hominivorax* are examined. Compared with PCR-RLFP, AFLP, or microsatellites, the easewith which RAPD-PCR can be used increases the number of samples that can be tested, thereby allowing an increased representation of populations in future RAPD analyses. Additionally, the use of 2 or more primers in RAPD reactions (Welsh and McClelland 1991; Micheli et al. 1993) could potentially increase the number of molecular markers that can be generated, thereby increasing the chances of developing RAPD ‘fingerprints’ for all populations and/or strains of *C. hominivorax*.

In conclusion, RAPD-PCR is a useful tool for interspecific, and promising for intraspecific, identification of *C. hominivorax*. This technique provides information helpful in the positive identification and monitoring of *C. hominivorax* in areas where the species is currently endemic, while its use does not demand a sophisticated laboratory. In case of suspected introduction, rapid and reliable identification allows eradication program officials to take appropriate steps to prevent further spread of the infestation (which can then lead to timely, appropriate corrective measures), but does not preclude the further verification of results from other laboratories or using other techniques. Aside from routine identification of field-collected immature samples and the potential for determination of sample origin, future applications of this technique may include checking suspected contamination in screwworm colonies in rearing facilities and assessing loss of genetic variability caused by selection and inbreeding in a colony. Moreover, developing RAPD markers that can distinguish males from females during early life stages would be valuable in the current plan of developing a male-only strain of *C. hominivorax* flies in mass-production facilities.

## Abbreviations

RAPD-PCR: random amplified polymorphic DNA-polymerase chain reaction
